# Science-based COVID-19 vaccine development

**DOI:** 10.1093/nsr/nwab193

**Published:** 2021-11-03

**Authors:** George F Gao

**Affiliations:** Institute of Microbiology, Chinese Academy of Sciences, China; Chinese Center for Disease Control and Prevention, (China CDC) China; Editorial Board Member of NSR

In late December 2019, a cluster of patients with pneumonia of unknown etiology (PUE) was reported [1]. Through the unbiased sequencing of bronchoalveolar-lavage fluid (BALF) from PUE patients and virus isolation, a novel betacoronavirus was discovered and named 2019-nCoV [2]. Later it was renamed SARS-CoV-2 by the International Committee of Taxonomy of Viruses (ICTV). It belongs to the subgenus sarbecovirus of genus betacoronavirus, family *Coronaviridae,* and is the seventh coronavirus that infects humans. On 30 January 2020, the World Health Organization (WHO) declared that the outbreak of coronavirus disease 2019 (COVID-19) constituted a Public Health Emergency of International Concern (PHEIC). Vaccines were urgently needed to prevent COVID-19. The complete SARS-CoV-2 genome was shared worldwide via GISAID immediately [1,3] and vaccine development was initiated even without obtaining the virus [3]. Frontier science has been playing a very important role in combating COVID-19.

According to the COVID-19 vaccine landscape announced by the WHO on 22 October, the 128 COVID-19 vaccines and candidates that entered clinical trials were based on seven strategies [4]: (i) Protein subunit vaccines. The predominant SARS-CoV-2 antigen, mainly spike (S) protein, is expressed, purified and adjuvanted as vaccines. Robust immunogenicity was achieved by rational design of antigens, such as a trimeric S protein with pre-fusion conformation (NVX-CoV2373) and tandem-repeat dimeric receptor binding domain (RBD) of S protein (ZF2001). Addition of novel adjuvants could elevate the induced cellular immune responses. This kind of design has the fewest viral components but with strong immunogenicity. (ii) Inactivated virus vaccines. The inactivated SARS-CoV-2 vaccines are safe and effective in humans, but a Biosafety Level-3 (BSL-3) facility is needed for large-scale culturing of the virus. China CDC converted its BSL-3 labs into small workshops for both SinoPharm and SinoVac to develop such inactivated vaccines in the end of January, 2020. It also contains other viral components in addition to the major immunogen of the S protein. (iii) mRNA vaccines. The mRNA is obtained from the transcription of a linear DNA template, and then purified and packaged into lipid-nanoparticles. The mRNA vaccines, such as Comirnaty (BNT162b2) and mRNA-1273, could induce both robust humoral responses and Th1-skewed cellular responses, and achieve high protection efficacy against COVID-19. However as this is the first time that mRNA products are being used for healthy populations, the long-term impact on humans needs further study. (iv) Virus-vectored vaccines. A SARS-CoV-2 antigen is inserted into recombinant virus vectors, especially adenovirus vector, and expressed in host cells upon inoculation. The vaccines AZD1222, Ad5-nCoV, Ad26.COV2.S and Sputnik V were approved for use and showed good immunogenicity. The pre-existing vector virus antibodies are a limited factor for booster jabs. (v) DNA vaccines. The SARS-CoV-2 antigen gene is inserted into recombinant plasmids. The production of DNA vaccines is very fast, compared to other types of vaccines. The clinical trials of INO-4800 showed good immunogenicity and safety. Recently, a DNA vaccine against COVID-19, ZyCoV-D, has been approved in India. (vi) Virus-like particle (VLP) vaccines. Structural viral proteins and VLP core proteins are co-expressed or combined to form particles without infectious ability as they lack the virus genome. VLP vaccines elicited immune responses like the real virions with high immunogenicity and safety. (vii) Live attenuated vaccines. Development of a safe and immunogenic attenuated virus vaccine usually takes a long period of time, and the risk of virulence reversion is of great concern.

COVID-19 is an unprecedented disaster in human society in recent history. To control the COVID-19 pandemic, four vaccines received conditional marketing authorization (CMA) in China, including BBIBP-CorV, CoronaVac and Ad5-nCoV. In addition, three vaccines received emergency use authorization (EUA) in China, including the subunit protein vaccine ZF2001—a dimeric RBD protein with aluminum hydroxide as adjuvant—which provided 81.76% efficacy against COVID-19 of any degree of severity in Phase III clinical trials.

Although inoculation with COVID-19 vaccines did not completely prevent SARS-CoV-2 infection, it did provide fundamental immunity in humans so far as reducing severe symptoms and death. A recent real-world study in Guangdong found that full vaccination of inactivated vaccines conferred adjusted effectiveness of 70% against pneumonia and 100% against severe illness caused by the Delta variant [5], suggesting the effectiveness of the vaccine in preventing the action of this SARS-CoV-2 variant.

Novel vaccines, such as mRNA and protein subunit vaccines, play a crucial role in the battle against COVID-19. In particular, mRNA vaccines were used for the first time in healthy populations and found to provide excellent protection. New vaccine technologies, such as mRNA vaccines, also bring hope for cancer immunotherapy, rare genetic disorders and other diseases. Looking back into the past, smallpox and rinderpest were eradicated by vaccination campaign. In addition, polio was eliminated in most countries and also nearly eradicated worldwide. With changes in climate, ecological environment and human behavior, novel pathogens will continuously emerge and re-emerge. The COVID-19 pandemic has taught us that science will lead the way for prevention and cure of infectious diseases. Scientists and governments around the world need to work together to share scientific information, medical resources and vaccine technologies. Basic research in virology and infectious diseases needs to be further strengthened in order to prepare the world for the future challenges of potential viral infections.
